# Interactive curriculum learning increases and homogenizes motor smoothness

**DOI:** 10.1038/s41598-024-53253-3

**Published:** 2024-02-03

**Authors:** Vaynee Sungeelee, Antoine Loriette, Olivier Sigaud, Baptiste Caramiaux

**Affiliations:** Sorbonne Université, CNRS, ISIR, Paris, France

**Keywords:** Computer science, Information technology

## Abstract

One of the challenges of technology-assisted motor learning is how to adapt practice to facilitate learning. Random practice has been shown to promote long-term learning. However, it does not adapt to the learner’s specific learning requirements. Previous attempts to adapt learning considered the skill level of learners from past training sessions. This study investigates the effects of personalizing practice in real time, through a curriculum learning approach, where a curriculum of tasks is built by considering consecutive performance differences for each task. 12 participants were allocated to each of three training conditions in an experiment which required performing a steering task to drive a cursor in an arc channel. The curriculum learning approach was compared to two other conditions: random practice and another adaptive practice, which does not consider the learning evolution. The curriculum learning practice outperformed the random practice in effectively increasing movement smoothness at post-test and outperformed both the random practice and the adaptive practice on transfer tests. The adaptation of practice through the curriculum learning approach also made learners’ skills more uniform. Based on these findings, we anticipate that future research will explore the use of curriculum learning in interactive training tools to support motor skill learning, such as rehabilitation.

## Introduction

Practice is known to play a fundamental role in motor learning, but the way practice should be structured remains unclear^1^. Research in motor learning has shown that adding variability during practice can facilitate motor adaptation and the learning of a new skill^2–4^. One motor learning phenomenon which has been studied to understand the effects of task switching on the organization of motor learning tasks is contextual interference (CI)^3^. According to the CI hypothesis, inducing variability in task order, e.g. through random practice, facilitates learning and transfer skills^5^. This theory has been tested in implicit learning contexts^6^, applied to sports, such as executing a volleyball serve^7^, practicing music (the left-hand interval leap on the piano)^8^ and learning piano sequences following a tempo change during practice^9^. However, randomly varying motor tasks induces high levels of CI and alters the nominal difficulty of the tasks without considering the skill level of the learner. Moreover, some research has demonstrated the negated effects of random practice in certain conditions—when the motor skill is complex^10,11^ and when the skill level is poor^12^.

In the motor learning domain, the relationship between task difficulty and performance has been studied, resulting in theories such as the challenge point framework^13^. It asserts that learners should face optimal challenges to enhance retention of skills. The optimal challenge point is expected to evolve throughout the learning process, as learners become more proficient at processing information. Consequently, this theory proposes that the drawbacks associated with random practice, such as its negative impact on immediate performance, can be overcome by tailoring practice schedules to the learner. In existing literature, researchers have explored the effects of varying the challenge by manipulating factors such as the amount of available information or suggesting different types of feedback^14,15^. Other studies have explored the efficacy of learner adaptive approaches^16–18^. A notable example is a study that introduced an algorithm capable of assessing the difficulty of tasks by analyzing the average performance errors made by learners in a preceding training session. The number of practice trials per task was adjusted accordingly, assigning more trials to tasks exhibiting lower performance. This study found that adjusting the number of trials based on task difficulty results in superior performance compared to random practice. The automatic adaptation of difficulty has also been explored in the realm of robot-assisted therapy for hand rehabilitation^19^. The rule which updated the challenge attempted to maintain the patient’s performance around a target level. It was shown that the difficulty adaptation algorithm effectively yielded performance levels close to the target. Subsequent research investigated how individualized challenge points affect learning^17^. Individualized challenge points (reaction time values) were determined from a previous study, where performance curves were derived for each participant. The goal was to investigate whether a high or low contextual interference was most beneficial for a pair-matching task using key presses. Even though they did not compare their approach to a random practice, they showed that a learner adapted practice, with high levels of contextual interference, improve motor learning and do not seem to have a significant negative impact on immediate learning. The findings from this research indicate that consistently evaluating motor skills and adapting the difficulty to the learner could enhance immediate performance, whereas increased contextual interference might aid in skill retention. Our objective in this paper is to investigate this particular scenario.

To tailor difficulty levels for individualized practice, automated techniques to assess performance have been created. These techniques evaluate performance through various measures of task difficulty, e.g. the learning progress on a task. Recent research in educational sciences has explored how practice can be adapted to the learner by building a curriculum of tasks in real time (curriculum learning), e.g. to cater for a time budget and to adapt to different types of learners^20^. A Multi-Armed Bandit (MAB) algorithm was used to propose tasks in an Intelligent Tutoring System. The multi-armed bandit (MAB) problem involves determining which lever of a K-slot machine to activate in order to optimize the cumulative reward over a series of trials. When applied to pedagogical action selection, each educational action is analogous to a lever in the MAB framework, and the objective is to maximize the advantages for the students. In this context, learning activities were proposed based on the current learning progress of each activity. At each time step, an activity providing significant learning progress is scheduled. Hence, a specific activity will cease to yield rewards or foster further learning progress once the student masters it. Since there was a large pool of activities to choose from, the algorithm was steered using a pedagogical graph of activities provided by an expert, thus reducing the number of activities to explore. The generated curriculum proved to be more effective than one crafted by an expert, and the learners exhibited greater motivation to complete the learning activities. In the cognitive science domain, other research has employed the MAB algorithm, e.g. to select actions in the form of feedback to present to learners while they are learning a gesture task^21^. In that work, the goal was to select feedback having a high probability of success (based on successive rewards previously obtained) on the gesture dimensions which were poorly performed. An experiment designed to compare a sequence of expert feedback with the feedback generated by the algorithm showed that performances were better when automated adaptive feedback was provided.

In this paper, we propose a curriculum learning approach, which aims to sample tasks providing high learning progress. This approach involves dynamically adapting the selection of tasks based on the estimated gain (computed as the learning progress) of scheduling a particular task. The task exhibiting the greatest learning progress may not be inherently easy; rather, it is the one currently enhancing the learner’s skills. For instance, once a task is mastered, its learning progress diminishes, rendering it less likely to be recommended in subsequent trials.

In this study, we investigate the potential benefits of using a curriculum learning approach to create variable, but learner-adapted practice. This strategy proposes tasks which provide the highest learning progress in real-time, using machine learning techniques. In our paper, we show how an automated adaptive schedule based on the principle of providing tasks with fast learning progress impacts performance. The selection of this metric is based on its efficacy in identifying tasks that are on the boundary between being too challenging and too straightforward to the learner^22,23^. To this effect, we adapted a Multi-Armed Bandit algorithm from previous work^20^, using a measure of the motor learning to compute learning progress.

In particular, our research aims to answer the following questions: how does the adaptation strategy during practice affect the learners’ (1) immediate performance, (2) delayed performance and (3) transfer capabilities? Previous work in adaptive learning pedagogy showed that proposing tasks which provide high learning progress can improve performance^20^. Furthermore, previous research in motor learning^17,18^ has shown that continuously assessing motor skills may improve immediate performance compared to a practice with a high level of contextual interference, e.g. a random practice. We hypothesized that by selecting tasks which provide high progress rates to the learner, the curriculum learning condition would help learners achieve better immediate performance after training than a random practice.

We conducted a between-subject experiment which lasted two days, where participants had to execute a motor task, adapted from previous work^24^. It involved performing wrist movements to drive a cursor through an arc channel of a certain width. We derived different nominal difficulty levels of the motor task by varying the width of the channel. The functional difficulty, i.e. the level of challenge offered at a given time, based on motor control capabilities, is learner-specific. On day 1, participants were tested before practice in a pre-test, where the task width was fixed. Practice involved performing tasks of different channel widths. After practice, a post-test with the same task parameters as the pre-test assessed immediate performance. On day 2, participants were tested in a delayed retention test. The retention test was composed of two parts: a test where the task was the same as the post-test; and transfer tests, where the channel diameter and movement time changed, but the width remained the same. To assess performance, we recorded the cursor coordinates of the participants on the screen while they carried out the motor task. The performance measure used to determine the learning progress was the proportion of movements which were within the channel [(called the In-Channel fraction (ICF)], bounded between zero and one. As a secondary metric, we evaluated the Movement Jerk^25^, which is a measure of the movement smoothness. The experiment was conducted under 3 conditions: (1) a random practice, (2) an error-adaptation practice adapted from previous work on learner-adapted learning^16^, where the number of trials was adapted based on performance errors on each task performed over a few trials at the start of the training phase and (3) a curriculum learning practice.

## Results

In order to designate each phase of the experiment, we use the following term: *Phase*, which was either Pre-test, Post-test, Retention or Transfer-x, where x is between 1 and 5 to designate different channel diameter and movement time. The specificities of each task are listed in Table 1.

**Table 1:** Phases of the experiment and the corresponding task specifications: movement time and channel diameter.

Phase	Movement time (s)	Channel diameter (px)
Pre-test	1.0	800
Post-test	1.0	800
Retention	1.0	800
Transfer-1	1.0	600
Transfer-2	1.0	400
Transfer-3	0.7	800
Transfer-4	0.7	600
Transfer-5	0.7	400

The movement onset and end were indicated by an audio signal. We began by analyzing whether movement time was comparable between participant’s performances per phase. We conducted a one-way ANOVA with Condition and relative Movement Time taken by participants assigned to each condition and there were no significant differences between conditions for the Post-test ($F(2,105) = 0.190$, $p = 0.826$), Retention ($F(2,69) = 0.572$, $p = 0.567$), Transfer-1 ($F(2,33) = 0.084$, $p = 0.920$), Transfer-2 ($F(2,33) = 0.340$, $p = 0.714$), Transfer-3 ($F(2,33) = 0.475$, $p = 0.626$), Transfer-4 ($F(2,33) = 0.034$, $p = 0.967$), Transfer-5 ($F(2,33) = 2.165$, $p = 0.131$). Thus, the conditions were comparable in terms of time taken to perform the movement.

### Assessment of the impact of task scheduling on immediate performance

During the pre-test, participants had to trace a trajectory in a 18 px wide and 800 px large channel. They were asked to try to execute the movement in the movement time indicated (MT = 1 s). Both the pre-test and post-test involved the same task.

**Figure 1:**
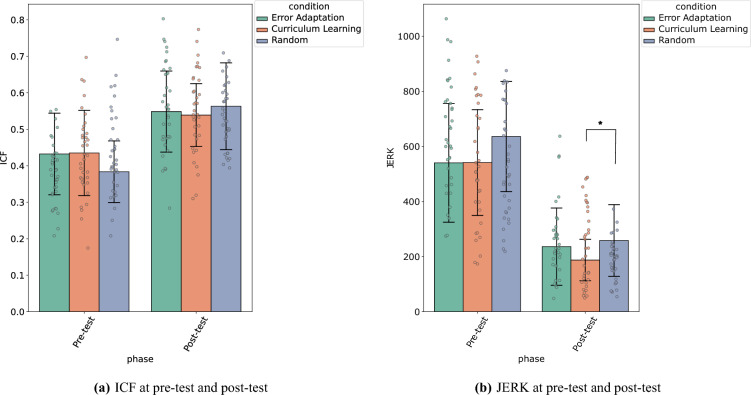
The two performance metrics, ICF and JERK, computed on day 1 for each practice condition, at pre-test and post-test for a task with channel diameter 800 px and movement time of 1 s. The error bars show the standard deviation of the ICF and JERK, respectively.

We began by assessing their In-Channel Fraction (ICF) performance, i.e. the portion of the executed trajectory that is traced within the channel at pre-test. Figure 1a shows the results. To analyze whether the initial performance between conditions is comparable, we conduct a a two-way ANOVA with practice Condition and Phase (pre-test and post-test) as independent variables and ICF as dependent variable. We found no significant difference in the mean ICF between the three conditions considered in the study (p = 0.60), but a significant effect of phase (pre-test, post-test) ($F(1,210) =83.2$, $p < 0.0001, \eta ^2=0.28$) and no interaction ($p=0.08$). In other words, the type of training does not seem to have an influence on the performance of the participants measured via the ICF. A T-test between the aggregated means for these two phases shows a significant difference ($p < 0.001$), where the means are $\mu _{ICF}=0.42$ at pre-test and $\mu _{ICF}=0.55$ at post-test. Therefore, performance increased between these two phases, confirming a motor learning mechanism in participants.

To investigate performance immediately after training, we also evaluated the movement smoothness of the gestures performed through the Movement jerk, the third derivative of the movement, integrated along the trajectory^24,25^. Figure 1b shows the results at pre-test and post-test. A two-way ANOVA with Condition and Phase (pre-test, post-test) as independent variable and Jerk as dependent variable shows an effect of condition ($F(2,105) = 4.60$, $p=0.01, \eta ^2=0.04$) and phase ($F(1,105) = 226.7$, $p< 0.0001, \eta ^2=0.52$) and no interaction ($p=0.42$). A post-hoc analysis with Tukey’s test (with 95% confidence level) at Pre-test shows that there is no significant difference in mean movement jerk between training conditions. A similar test performed at post-test shows that the mean JERK for the curriculum learning condition is significantly lower than the random condition ($p=0.03$). On the other hand, there is no significant difference between the random and error-adaptation condition ($p=0.71$). Thus, participants of the curriculum learning condition demonstrated smoother movements at post-test than those of the random condition.

### Assessment of the impact of task scheduling on retention and transfer tests

In order to assess learning at retention and transfer to novel tasks, we tested participants on day 2. Day 2 tests are split into one retention test, where the task parameters were the same as those of the pre-test and post-test (i.e. a channel of width 18 px, diameter 800 px, and movement time 1 s), and 5 transfer tests, where the diameter of the channel and movement times were modulated while the channel width remained constant. In particular, each transfer test was a combination of a unique diameter (D) and movement time (MT) as follows: (D = 600 px , MT = 1 s), (D = 400 px, MT = 1 s), (D = 800 px, MT = 0.7 s), (D = 600 px, MT = 0.7 s), (D = 400 px, MT = 0.7 s).

**Figure 2:**
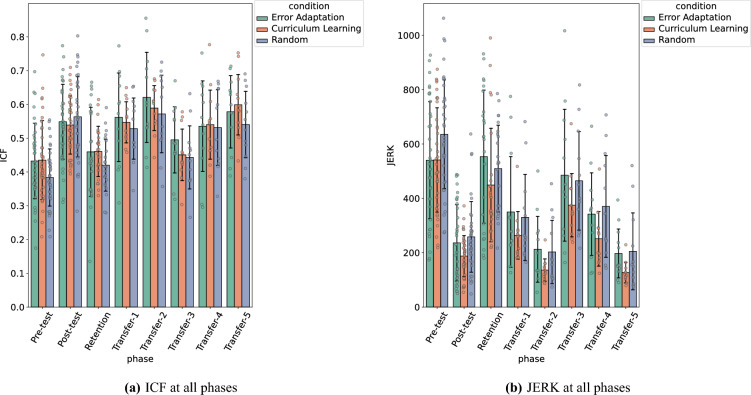
Performance metrics, ICF and Jerk, computed on day 1 and day 2 (pre-test, post-test, transfer, retention) per condition. The error bars show the standard deviation of the ICF and the JERK respectively.

First, we analyze whether retention performance differs across conditions. An ANOVA with Condition as the independent factor and ICF as the dependent variable shows no significant difference. In other words, the type of condition has no influence on ICF performance at retention. If we study the difference in performance between the pre-test, post-test, and retention phases with a one-way ANOVA, we observe that the phase has a significant impact on ICF ($F(1,105) = 46.2$, $p<0.0001, \eta ^2=0.25$). Tukey’s test shows that ICF values are higher at post-test than pre-test ($p<0.001$) and retention ($p<0.001$) but there is no difference in ICF between retention and pre-test ($p=0.16$), see Fig. 2a.

For the transfer tasks, we analyze whether performance in terms of ICF depends on the conditions and the type of transfer. A two-way ANOVA with Condition and Transfer (transfer tasks 1–5) as factors and ICF as the dependent variable shows a significant effect of the type of Transfer on ICF ($F(1,105) = 14.9$, $p<0.0001, \eta ^2=0.24$) but not the Condition ($p=0.07$). Tukey’s test shows that ICF does not significantly vary when the movement time decreases from 1.0 to 0.7, but significantly varies between transfer tasks where the diameter changes from 800 to 400 px ($p<0.001$ for each pairwise comparison) and from 800 to 600 px ($p<0.01$ for each pairwise comparison), but does not vary when diameter changes from 600 to 400 px.

A similar analysis taking the Jerk of the movement as the dependent variable shows no significant difference between the conditions on the value of the average JERK at retention ($p=0.24$) . Moreover, if we compare the average JERK values of the pre-test, post-test and retention phases using a one-way ANOVA, we observe that phase has a significant effect on the JERK value ($F(1,105) = 108.1$, $p<0.0001, \eta ^2=0.44$) (see Fig. 2b). Tukey’s test shows that the JERK value is significantly lower at post-test than pre-test ($p<0.001$) and retention ($p<0.001$). And the movement jerk is also lower at retention than at pre-test ($p=0.037$). Thus, the type of practice condition has no influence on the ICF or the JERK at retention, and the performance at retention is comparable to the performance at pre-test for ICF but better for the movement jerk.

For the transfer tasks, a two-way ANOVA with Condition and Phase (Transfer tasks 1–5) as factors and Jerk as the dependent variable shows that these two factors also have a significant impact on the Jerk ( $F(1,105) = 28.7$, $p<0.0001, \eta ^2=0.38$ for phase, $F(1,105) = 7.1$, $p<0.01, \eta ^2=0.06$ for Condition) and no interaction ($p=0.99$). A post-hoc analysis using Tukey’s test shows that there is no difference in terms of movement smoothness between the random condition and error adaptation condition ($p=0.89$) but movement smoothness is significantly lower for the curriculum learning condition ($p=0.014$ for the comparative test with the error- adaptation condition and $p=0.047$ with the random condition). In addition, movement smoothness also significantly varies when the diameter varies ($p<0.01$ for changes between 800 and 600 px or 400 px and $p<0.05$ for changes between 600 and 400 px, independently of movement time). Thus, we obtain smoother movements for the curriculum learning condition than the random condition and the error-adaptation condition at transfer and a decrease in movement smoothness for longer trajectories. These results are shown in the right panel of Fig. 2.

**Figure 3:**
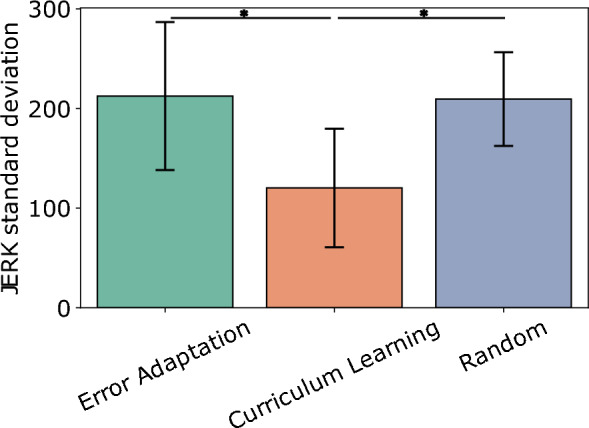
Variability of the JERK for each practice condition. The error bars show the standard deviation of the JERK standard deviation.

Finally, in Fig. 2 we observe a difference in jerk variance between conditions for the test phases. Thus, we plot the standard deviation of JERK performance across participants for each condition. Specifically, we compute standard deviations of trials for each condition and phase (post-test, retention and transfer) across conditions. A one-way ANOVA with Condition as the independent variable and Standard deviation as the dependent variable shows that the condition has a significant impact on the standard deviation of the JERK ($F(2,18)=4.97, p<0.019$). Tukey’s post-hoc test shows that the curriculum learning condition leads to a lower JERK standard deviation than the error adaptation condition ($p=0.029$) and, and the random condition ($p=0.035$) see Fig. 3. Thus, the curriculum learning condition has the smallest jerk variability (M = 121.22, SD = 49.55) compared to the error adaptation (M = 216.34, SD = 82.79) and random conditions (M = 202.91, SD = 43.46).

### Investigating learning schedules and rates

**Figure 4:**
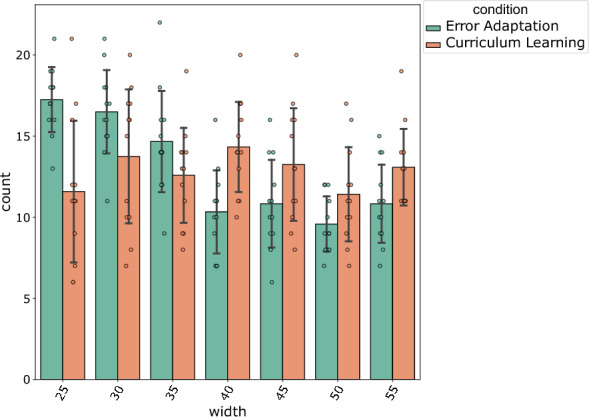
Frequency of tasks during training for curriculum learning and error adaptation conditions. The error bars show the standard deviation of task frequency.

To study the task schedules for each condition on learning, we counted the blocks of each width (25–55 px) allocated in the two conditions: error adaptation and curriculum learning (the random condition leading, by design, to an equal number of task trials per width). Figure 4 depicts the results, with the count of the number of blocks on the y-axis and the task width on the x-axis. We analyzed whether the number of task trials differed between width for each condition. We conducted a two-way ANOVA with Channel Width and Condition as independent variable and Count as dependent variable. We found a significant effect of width on count ($F(1,164) = 27.6$, $p<0.001, \eta ^2=0.14$) and a significant interaction between width and condition $F(1,164) = 29.1$, $p<0.001, \eta ^2=0.15$. Therefore, we applied pairwise t-test between condition for each width (with Bonnferoni correction) and found that there were significantly more trials at width 25 px in the EA condition compared to CL condition ($p=0.0013$) and there were significantly more trials at width 40 px in the curriculum learning condition compared to the error-adaptation condition ($p=0.0019$). This means that on average, compared to the participants of the error-adaptation condition, the participants of the curriculum learning condition practiced a larger number of low and intermediate difficulty tasks. However, a similar analysis at the start of training (first 10 blocks) shows no difference between the two conditions either in terms of Channel Width ($p = 0.39$) or Condition ($p = 0.94$).

**Figure 5:**
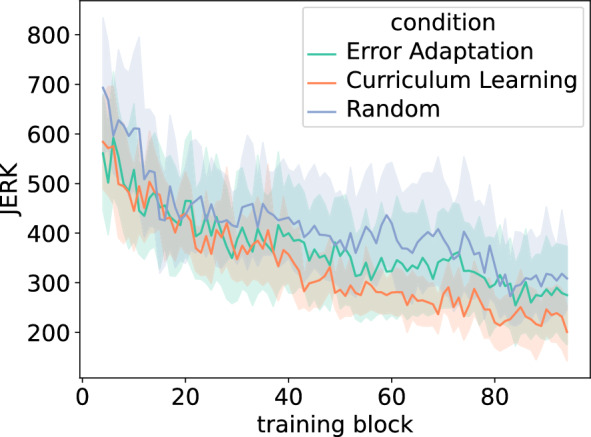
Average JERK over training blocks. The error bars show the standard deviation of the JERK.

To investigate how movement smoothness evolved during training, we computed JERK over the training blocks (blocks 4–94). Figure 5 shows the asymptotes of movement jerk during training. Performing an ANOVA yields a significant difference between conditions (p < 0.001) in the final 10 training blocks. Tukey’s post-hoc tests show that curriculum learning has lower JERK values over the last 10 blocks of training compared to the error adaptation ($p=0.013$) or random condition ($p<0.0001$).

To investigate the level of contextual interference for each condition, we computed the number of task switches for each participant of each condition. An ANOVA with Condition and Number of switches shows a significant influence of Condition. A post-hoc analysis using Tukey’s test shows that the curriculum learning condition has a lower number of switches than the random condition ($p<0.01$) as well as the error adaptation condition ($p<0.01$). There were no significant differences between the other two conditions.

**Figure 6:**
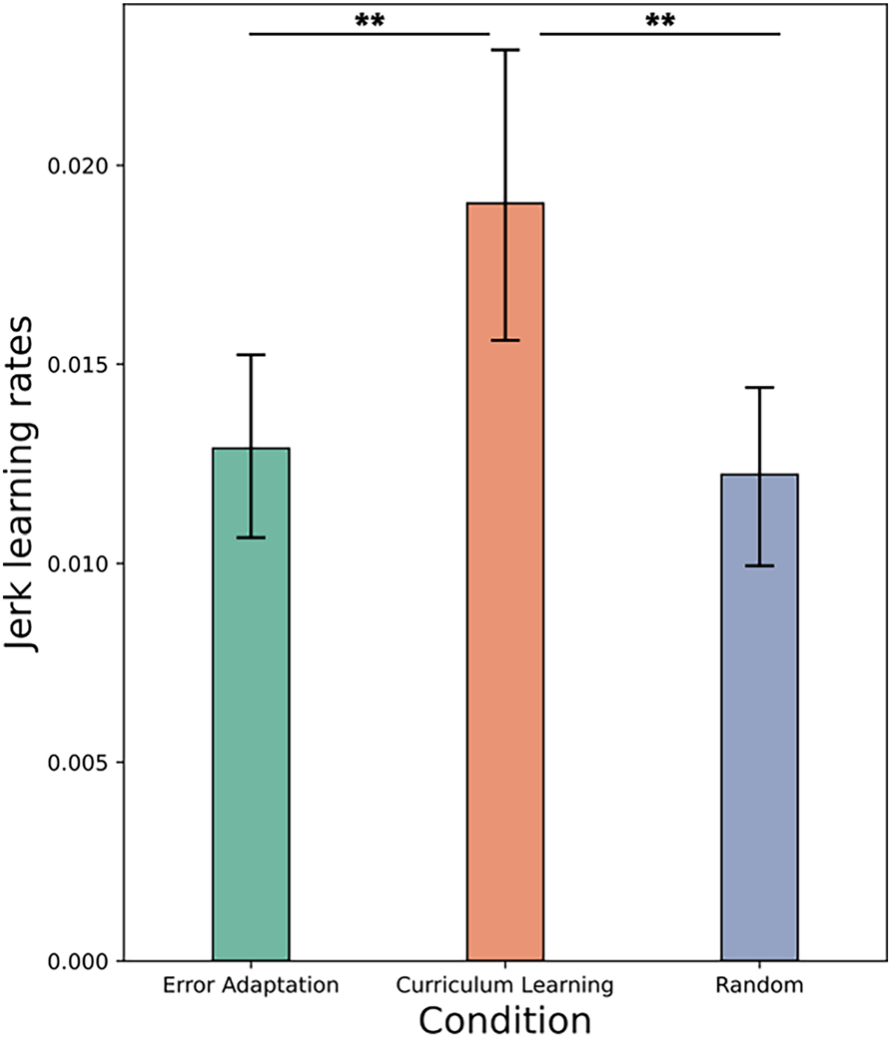
Learning rates for the JERK across conditions. The error bars show the standard deviation of JERK learning rate.

Finally, we inspected the learning rates for each condition. To measure the learning rate of each task during practice, we performed a linear regression of the log-transformed ICF and Jerk values. An ANOVA with Condition as factor and ICF learning rate as the dependent variable shows no significant difference. Thus, neither the type of task nor the practice condition had an impact on the rate of learning for the ICF. We then conducted a similar test with Jerk learning rate as the dependent variable and it shows that there is a significant impact of Condition ($F(2,231)=6.55, p=0.0017, \eta ^2=0.05$). A post-hoc analysis using Tukey’s test shows that learning rates for curriculum learning condition is significantly higher than for both the random condition ($p=0.003$) and for the error adaptation condition ($p=0.009$). Figure 6 reports the mean learning rates per condition together with their confidence interval.

## Discussion

The main aim of the present study was to investigate the effects of adapting the task difficulty based on a performance metric commonly used in psychology and human behaviour studies: the learning progress, on a movement learning task. We compared three strategies, a random practice and two algorithms which adapted practice to the skill level of the learner: (1) error adaptation, which schedules a larger number of tasks with the worst performances and (2) curriculum learning, which schedules tasks which currently provide a larger increase in performance. Our results showed that the adaptive practice conditions yielded comparable performances to the random practice when evaluated immediately after practice, when computed through the In-Channel Fraction (ICF) at post-test. On the other hand, participants assigned to the curriculum learning condition demonstrated smoother movements, computed as the movement jerk, at post-test than those assigned to the random condition. Executing smoother movements after practice can be seen as a result of trajectory optimization, which leads to skill acquisition^24,26^. These results suggest that participants assigned to the curriculum learning condition reduced the effort required to perform the movement and thus improved their movement efficiency compared to a random practice. The lower JERK performance of the adaptive conditions compared to the random condition suggests that adaptation of the difficulty based on skill level could be an important factor for decreasing movement effort and improving movement efficiency. In general, these results are in line with findings of other studies on learner–adapted practice^16,18^, i.e. some aspect of performance is better when practice is adapted to the learner than when tasks are scheduled randomly.

While the adaptive schedules were based on the ICF metric, we found differences between conditions only when we assessed the JERK and not the ICF. Further analysis of the JERK and the ICF measures showed a weak negative correlation ($r=-0.32$, $p<0.05$). This could mean that participants favored a smoother trajectory over remaining inside the channel. To better understand the implications of these results, future work should investigate the movement smoothness as a metric for motor learning. In particular, it would be interesting to investigate what strategy participants used to improve movement smoothness, e.g. learning to trace the arc shape and then progressively increasing the smoothness or the other way round. Results for retention, on day 2, showed that the type of practice did not have an effect on either the ICF or the JERK. Our interpretation is that this effect could be due to our choice of test task, which was more complex than those presented during the practice. According to the specificity of practice view^27^, similarity between practice conditions and test conditions is an important factor for successful performance. A larger learning effect and differences between conditions could have been observed with a target task at the same level of complexity as those presented during practice. However, testing on a task which has been seen during training would imply that some participants would, on average, practice this task more often than desired between training phases. A longer delay between training and testing could resolve this problem. In addition, although benefits of adaptive practice compared to random practice in terms of retention have not been observed in this study, the advantages of random practice are less unanimous in applied tasks^10,11^. This calls for more investigations on adaptive learning in these settings. We tested transfer capabilities on the next day by modulating either the diameter of the channel or the movement time, or both. While differences between conditions were not observed for the ICF, there were differences in JERK performance. In particular, the curriculum learning condition outperformed the other conditions. There was no difference between the other two conditions. According to the structural learning theory, inducing variations randomly during training of a motor task facilitates learning of other motor tasks sharing the same structure as the original^28^.Our results show that an adaptive strategy has the potential to improve the transfer capabilities of learners.

The results also showed that the curriculum learning participants learned to increase the movement smoothness faster than the other participants. Past research in the cognitive domain^20^ showed that skill acquisition was faster for participants who learned under a schedule which optimized the learning rate. Similarly, our findings suggest that a faster learning rate could also be achieved when using a learning rate metric for optimization in a motor learning context. Remarkably, the JERK standard deviation for participants assigned to the curriculum learning condition was significantly lower than the error adaptation and random condition. This suggests that curriculum learning leads to more consistent performances across participants in terms of movement smoothness. This reinforces the strength of the algorithm to optimize task difficulty, independently of the learner. We also found that on average, participants assigned to the error adaptation condition trained on tasks of higher difficulty than the curriculum learning condition and switched exercises more often (higher CI). However, curriculum learning yielded comparable (ICF at post-test, retention, transfer) or better performances (JERK at post-test, transfer) to error-adaptation. If practicing tasks of lower difficulty level do not penalize motor learning, this type of practice could also be more motivating to learners, making it an attractive candidate for a customizable interactive motor learning system. This is supported by the fact that participants considered the schedules of the curriculum learning condition to be appropriate for learning, as reported in a questionnaire after training.

## Conclusion

In this paper, we have described a study that focuses on facilitating motor learning in an interactive setting through an adaptive algorithm based on Multi-Armed Bandits (MABs). The results suggest that this adaptation could be promising to facilitate learning characterized by an increase in the smoothness of the movement along its trajectory, as well as to take into account inter-individual differences to standardize performances. These results show the interest of a real-time approach to create a curriculum. Thus, this article provides a theoretical understanding of the interest of curriculum learning for motor learning and highlights a promising direction for studies on learner adapted practice.

## Method

All experimental procedures were approved by the Ethical Research Committee of Sorbonne university. All participants gave informed consent prior to participating in the study. The experiment was performed in accordance with relevant guidelines and regulations.

### Participants

We recruited 36 participants, among which 21 identified as male, 14 as female and 1 as a non-binary. We counted 3 left-handed and 33 right-handed participants. The participants were on average 18–24 years old. They reported not having any motor or cognitive disabilities. They were recruited within Sorbonne University, through a mailing list, or were invited to participate on campus. Prior to participation, the participants signed a consent form and could ask to stop the experiment any time.

### Apparatus

**Figure 7:**
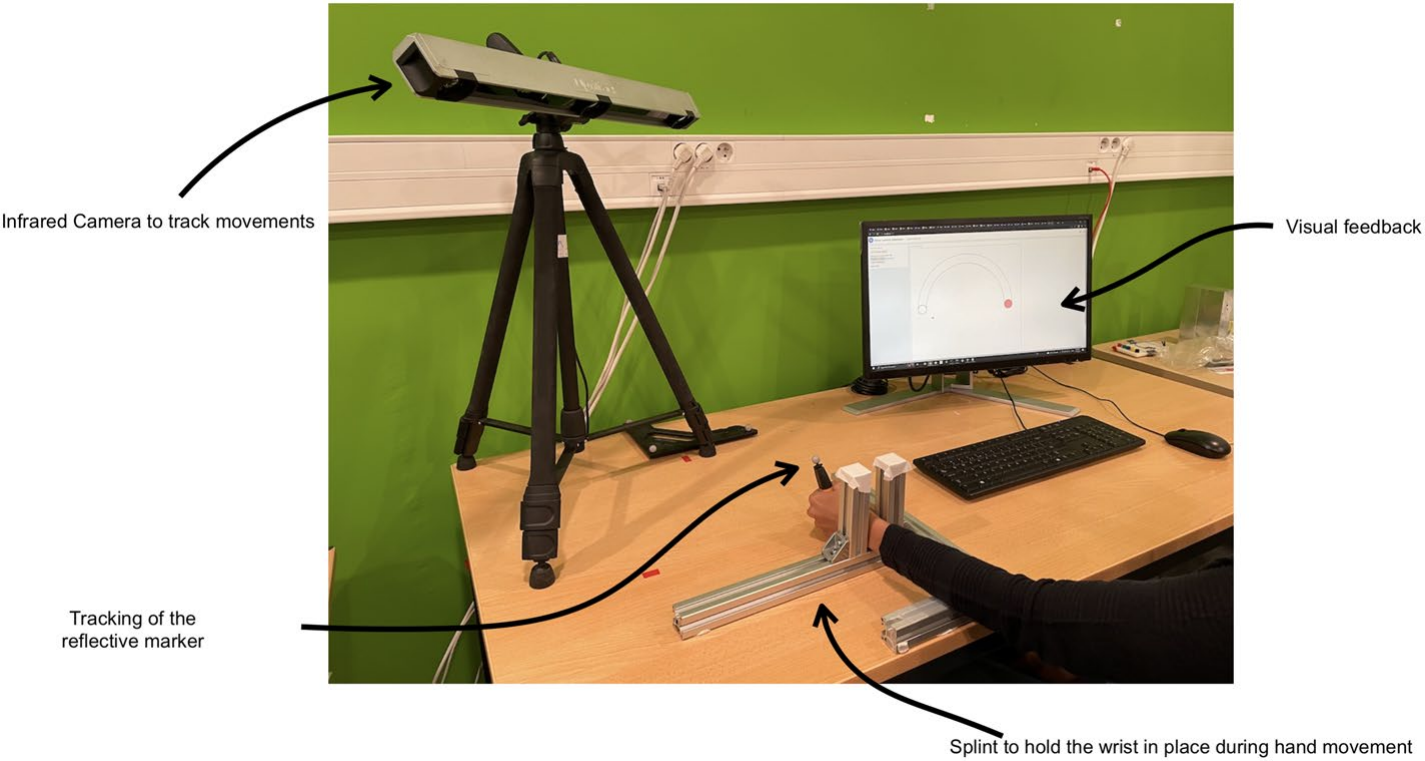
Apparatus used for the study. The participant’s forearm is maintained in a splint. The participant holds a marker in the hand, that is captured by an infrared camera. The marker controls a cursor displayed on a screen.

Figure 7 illustrates the experimental setup. The participants are seated at a table equipped with a motion capture system and a screen. Participants place their non-dominant hand through a splint, which was adapted to the size of their wrist for comfort. The splint is used to limit the movements of the forearm of the participant to pronation and supination to add novelty to the task. The participants are also equipped with a cursor in the form of a marker pen, on which is placed a reflective marker. With the marker in the hand, the participant has to trace a trajectory on screen while trying to maintain the latter inside the channel. The participant’s movement is captured by an infrared camera (Optitrack v120) and is drawn on screen to provide visual feedback after each trial. The reflective marker and the position of the cursor on screen are made to correspond by projecting the marker on a plane perpendicular to the participant’s forearm. The origin is considered to be the rest position of the participant, and coordinates are scaled to pixel values so that moving from one side of the channel to the other is in the order of one centimeter. The screen has a refresh rate of 144 Hz. Finally, an audio signal indicates to the participant that the allocated movement time is over. We carried out the experiment on a dedicated machine and use the Marcelle library^29^ to implement the JavaScript application, which is launched via a web browser.

### Task

**Figure 8:**
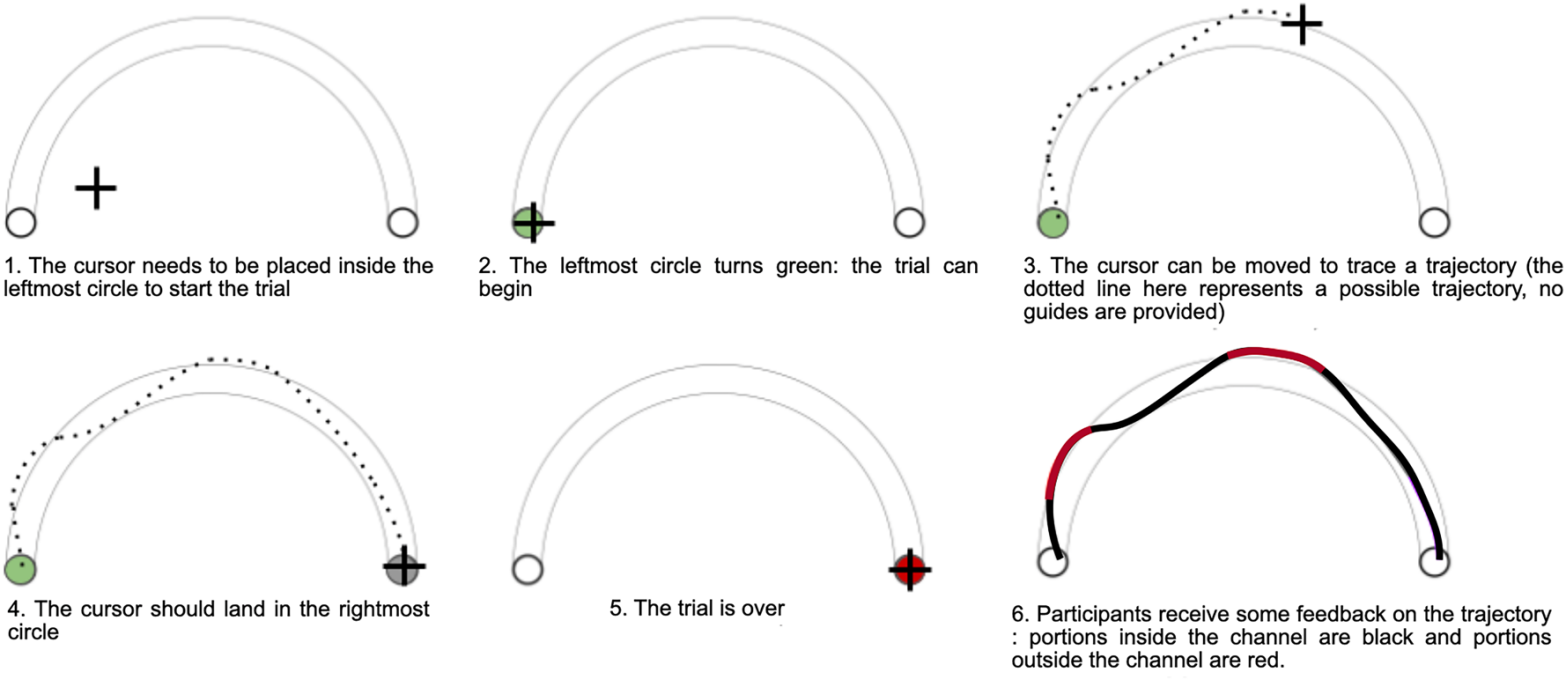
The steps involved in a trial of the experiment. After the leftmost circle turns green, the participant can begin to trace the trajectory using the cursor. The participant tries to keep the trajectory within the channel and which stops in the rightmost circle. The visual feedback of the trajectory is represented by a red trace if the trajectory is outside the channel and black otherwise.

**Figure 9:**
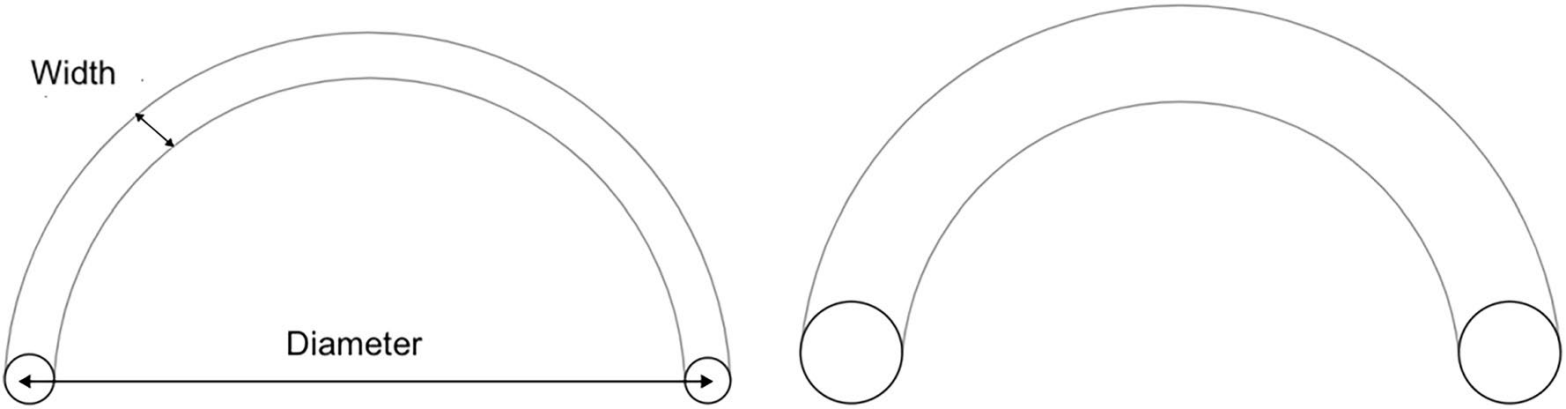
Two examples of the task seen during training: the width of the channel was modulated to change the difficulty.

The chosen motor task involves performing wrist movements with a cursor while keeping the projected 2D trajectory of the cursor inside a channel of a certain width, which appeared on a screen. Figure 8 shows the task used for the experiment. Wrist movements control a cursor visualized on a screen placed in front of the participant. At the start of each trial, the participant places the cursor inside the left circle of the arc-channel (Fig. 8, 1.). Once inside the small circle, an audio as well as a visual signal (the circle turns green) indicates to the participant that he/she can start to perform the gesture (Fig. 8, 2.). The participant drives the cursor inside the right circle through a wrist movement, and there is no visual feedback on the trajectory prior to reaching the right circle (Fig. 8, 3., 4.). Once the right circle is reached, the participant receives visual feedback on the quality of the trajectory: the trace of the trajectory is black if it is inside the channel and red otherwise (Fig. 8, 6). Performance scores are not displayed during the experiment. Movement time, designated as MT in this paper, is constant; the start and end times of the movement are indicated by an audio signal. To change the difficulty of the task, we vary the channel width (Fig. 9), while the length of the channel remained constant. This task is inspired from previous research in motor control^24^. In this work, participants have to learn to perform wrist movements along the x and y axes and the movements are displayed on screen in 2D. To limit exploration of redundant degrees of freedom during learning, the participant’s wrist is constrained by a splint, which limits the movements of the forearm.

### Conditions

In this section, we present the three conditions tested in this study. These conditions concern the scheduling of motor tasks during the training phase. In the next sections, these conditions will be referred to as Condition, to connote the type of training.

#### Curriculum learning (CL)

This condition implements the scheduling algorithm based on previous work^20^. It uses a Multi-Armed Bandit (with $K = 7$ arms) to select tasks which are likely to produce the best learning progress. The reward is the learning progress, computed as the absolute difference between the performance score of the previous and current (consecutive) blocks of the same task. Hence, the reward adapts in real time to the performance of the learner. A *SoftMax* strategy is used to favor the selection of tasks having a larger learning progress. Formally, we can write the algorithm as follows, where *t* is the number of trials completed; *K* is the number of tasks; $\textbf{w}_t$ is the estimated reward for each task; $\gamma ,\beta ,\eta$ are the update weights. The steps for selecting tasks are described below. The update in step 4 tracks the estimated reward of the scheduled task. The parameters $\beta$ and $\eta$ balance the importance given to the previous estimate and the actual reward (learning progress) obtained for the scheduled task. After a pilot test, we found that an exploration parameter value $\gamma$ = 2 balances exploration and exploitation. The values of $\beta$ and $\eta$ were set to 0.5, to weigh estimated rewards and actual rewards equally. The weights of the Softmax function are set to 0.1 for all tasks to ensure small updates of the estimated rewards.

- *Step 1.* The vector $\textbf{w}_t$ is initialized.
- *Step 2.* The probability distribution P_*i*_(t) of task *i* at time *t* is computed as follows:
  $$\begin{aligned} P_i(t) = \frac{exp(\textbf{w}_i(t)^\gamma )}{\sum _{i=1}^{K} exp(\textbf{w}_i(t)^\gamma )} \end{aligned}$$
- *Step 3.* A task is chosen according to the distribution *P*(*t*). The reward *r* is the absolute difference between the last *d* trials and the previous *d* trials of the same task. $ICF_i$ is the ICF performance on a task at time *i*.
  $$\begin{aligned} r = \sum _{i = t-d}^{t} \frac{ICF_i}{d} - \sum _{i = t - 2d}^{t-d} \frac{ICF_i}{d} \end{aligned}$$
- *Step 4.* The vector $\textbf{w}_i$ is updated:
  $$\begin{aligned}\textbf{w}_i = \beta \textbf{w}_i + \eta r\end{aligned}$$

#### Error adaptation (EA)

This condition is an adapted version of an algorithm from the computer-controlled learning literature^16^, which schedules tasks with the worst performances by adapting the number of trials. The performance error is computed from a pre-test, since the errors computed from a few trials during learning can have a high variability. It is therefore not a real time adaptation.

For each task, the number of trials for training is determined in a pre-test according to the performance error (Error = 1 − performance). To avoid allocating too many trials to one task, a minimum and maximum proportion of trials is fixed. We adapt these limits to the number of tasks in our experiment by multiplying the proposed proportions of Choi et al.^16^ by a factor of 4/7, where 4 is the number of tasks in Choi et al.’s experiment. Hence, we come up with these numbers: minimum proportion of trials = $7.15\%$ and maximum number of trials = $36.3\%$. The number of trials is adjusted according to the performance error while satisfying the above proportions: $N_i = \text {min}(\text {max}(N * E_i, N * 0.0715), N * 0.363)$, where $N_i$ is the number of trials for the task *i*, and *N* is the total number of trials for all tasks, $E_i$ is the normalized performance error for the task *i*. The trials are then evenly distributed among the blocks of the experiment to decrease the chances of consecutive trials of the same task.

#### Random

This condition is the baseline commonly used in the literature^13^ to illustrate the advantages of contextual interference for practice schedulesƒ. The 84 blocks (each block consisting of 4 trials each) were pseudorandomly shuffled for each participant.

### Design and procedure

The participants take part in two separate sessions on two consecutive days: *training* (day 1) and *retention-transfer* (day 2). The two sessions are separated by 24 h. We adopt a between-subject design with three conditions. We assign the participants randomly to one of the three conditions so that each condition had 12 participants.

**Figure 10:**
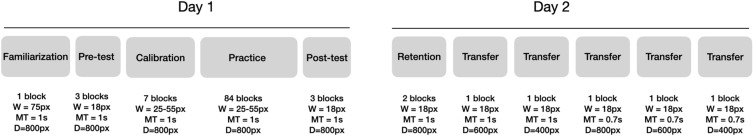
Experimental procedure. The first session (day 1) lasted 45 min on average and consisted of 5 phases: familiarization, pre-test, calibration, training and post-test. All blocks of day 1 comprised of 4 trials. The second session (day 2) lasted 15 min and comprised 2 phases: retention and transfer.

The first session (day 1) lasts 45 min on average and consists of 5 phases: familiarization, pre-test, calibration, training and post-test. All blocks of the first session have 4 trials. The second session lasts 15 min and had 2 phases: retention tests and transfer tests. All the blocks of day 2 have 12 trials. Figure 10 illustrates the procedure. We describe the different phases below.

- Familiarization: This single block task is created to help participants get familiar with the task. It involves tracing a trajectory in a channel which is 75 px wide and has a diameter of 800 px, within 1 s (MT = 1 s).
- Pre-test: A pre-test is included to measure the baseline performance of participants before training. It comprises 3 blocks of 4 trials and participants have to trace a trajectory in a channel which is 18 px wide and has a diameter of 800 px, MT = 1 s.
- Calibration: This phase has 7 blocks and is included to ensure that participants trained on all the 7 widths of the channel, irrespective of the scheduling algorithm. This is necessary for the *Error adaptation* condition, where the tasks to be scheduled depends on the performance on each task during the pre-test phase.
- Training: The training phase consists of 84 blocks of 4 trials each. The tasks involved arc channels of the following widths: 25, 30, 35, 40, 45, 50 and 55 px. The length (diameter) of the channel is fixed to 800 px during this phase, and MT = 1 s. The practice schedule depends on the condition assigned to the participant.
- Post-test: The post-test consists of 3 blocks of the same task seen during the pre-test. The goal of this phase is to verify the effect of each condition on performance.
- Retention: Retention tests are delayed performance tests of 2 blocks, with 12 trials each, conducted 24 h after training. The task parameters are the same as those of the pre-test and post-test, i.e. a channel which is 18 px wide and has a diameter of 800 px with MT = 1 s.
- Transfer: The Structural Learning theory^28^ posits that inducing variations randomly during training of a motor task facilitates learning of other motor tasks sharing the same structure as the original. The goal of this phase is to test how variations induced by the training condition affect the generalization of skills to similar tasks. For this phase of 5 blocks each consisting of 12 trials, we change the length (diameter) of the channel and the movement time indicated to participants to complete a trial. The width remains constant. Each block is a combination of a unique diameter (D) and movement time (MT) as follows: Block 1: D = 600 px et MT = 1 s; Block 2: D = 400 px, MT = 1 s; Block 3: D = 800 px, MT = 0.7 s; Block 4: D = 600 px, MT = 0.7 s; Block 5: D = 400 px, MT = 0.7 s.

### Data analysis

In order to analyze the movement data, we chose two metrics: the portion of the trajectory which is inside the channel (*In-Channel Fraction* (ICF)) and the smoothness of the movement *Movement jerk* (Jerk)). Participants are told that these two measures were equally important, and are instructed to perform smooth movements and try to remain within the channel for all trials of the experiment.

- Performance analysis (ICF): The performance measure is the In-Channel fraction (ICF), defined as the proportion of movements in the channel, bounded between 0 and 1. Hence, ICF values close to 0 indicates that the movement was poor, while values close to 1 indicates good performance. Performance error is computed as the complement of the ICF. To compare the effect of practice on trajectory, the block mean and variance are computed (over all trials in a given block) per participant before and after practice. This allows comparison of mean trajectory and trajectory variability at pre-test and post-test.
- Movement analysis (JERK): According to the minimum-jerk model hypothesis^25^, the motor system aims for maximum smoothness of end point movements and hence the jerk should be minimal when a motor skill is acquired. Following this model, we expect the movement smoothness to increase after training. This metric is regularly used to evaluate motor learning (e.g.^9,24^). To filter the raw data, we applied a third order Savitzky-Golay filter of degree 3 polynomial (taken from the Scipy signal python library (https://docs.scipy.org/doc/scipy/reference/generated/scipy.signal.savgol_filter.html) to each of the x and y position data respectively. The jerk is then computed as the sum of the squares of the filtered data along each dimension to obtain a single value.

## Data Availability

The datasets used in this study are available on Zenodo: https://doi.org/10.5281/zenodo.7824331.
